# Association between retinal vein occlusion and an increased risk of acute myocardial infarction: A nationwide population-based follow-up study

**DOI:** 10.1371/journal.pone.0184016

**Published:** 2017-09-12

**Authors:** Yu-Yen Chen, Shwu-Jiuan Sheu, Hsiao-Yun Hu, Dachen Chu, Pesus Chou

**Affiliations:** 1 School of Medicine, National Yang-Ming University, Taipei, Taiwan; 2 Department of Ophthalmology, National Yang-Ming University Hospital, Yilan City, Yilan County, Taiwan; 3 Community Medicine Research Center and Institute of Public Health, National Yang-Ming University, Taipei, Taiwan; 4 Department of Ophthalmology, Kaohsiung Veterans General Hospital, Kaohsiung, Taiwan; 5 Department of Education and Research, Taipei City Hospital, Taipei, Taiwan; 6 Deputy Superintendent, Taipei City Hospital, Taipei, Taiwan; Centre National de la Recherche Scientifique, FRANCE

## Abstract

**Objective:**

To investigate a possible association between retinal vein occlusion (RVO) and an increased risk of developing acute myocardial infarction (AMI).

**Design:**

A population-based retrospective cohort study using the entire population of the Taiwan National Health Insurance Research Database (NHIRD) from 1^st^ January, 2001 to 31^st^ December, 2013.

**Methods:**

A total of 37921 subjects with RVO were enrolled in the RVO group, and 113763 subjects without RVO were enrolled in the comparison group. The comparison group consisted of randomly selected individuals who were propensity score (PS)-matched with the RVO group at a ratio of 1:3, based on age, gender, obesity, stroke, hyperviscosity syndrome, glaucoma, and the use of antithrombotic drugs. A log-rank test was used to compare the cumulative hazard of AMI between the two groups. A multivariate Cox regression analysis was used to estimate the adjusted hazard ratios (HRs) of AMI, adjusted for PS, diabetes, hypertension, hyperlipidemia, congestive heart failure, and chronic renal failure.

**Results:**

The mean age of the cohort was 62.4±13.2 years. RVO patients had significantly higher proportions of diabetes, hypertension, hyperlipidemia, congestive heart failure, and chronic renal failure than the comparisons. The mean follow-up period was 5.52 years in the RVO group and 5.55 years in the comparison group (*p* = 0.16). A log-rank test comparing the cumulative hazard curves of AMI for the two groups revealed a significant difference (*p*<0.0001). In the multivariate Cox regression after adjustment for PS and confounders, the RVO group had a significantly higher risk of AMI (adjusted HR = 1.21; 95% CI: 1.13 to 1.30). When the RVO group was divided into central retinal vein occlusion (CRVO) and branch retinal vein occlusion (BRVO) and analyzed separately, both groups had significantly higher adjusted HRs for developing AMI than the comparison group. Moreover, the CRVO group had a significantly higher risk of AMI than the BRVO group.

**Conclusions:**

People with RVO are at significantly greater risk of developing AMI than individuals without RVO.

## Introduction

Retinal vein occlusion (RVO) results from thrombosis in the retinal vein, due to compression by a nearby atherosclerotic artery or increased blood viscosity. [1–4] Risk factors of RVO include hypertension, diabetes, hyperlipidemia, and older age. [5–10] All of these factors are also risk factors of acute myocardial infarction (AMI). Because RVO and AMI share a common pathogenesis of atherosclerosis and similar risk factors, these conditions might be associated.

Previous hospital-based studies investigating the relationships between RVO and the risk of developing AMI revealed inconsistent results. [5,10–14] Recently, follow-up studies based on healthcare claims databases were conducted to solve the problem but were also inconclusive. Hu et al utilized the Taiwan National Health Insurance Database (NHIRD) and found that RVO did not independently increase the risk of developing AMI during the 3-year follow-up period. [15] Werther et al analyzed claims data in the US (including Medicare and Medicaid) and showed that RVO was not significantly associated with AMI development during the 4-year follow-up period. [16] However, in South Korea, Rim et al used a database of a nationwide sample cohort and showed that RVO increased the risk of developing AMI [hazard ratio (HR) = 1.25; 95% confidence interval (CI): 1.02 to 1.52] during the 11-year follow-up period. [17] The inconsistent results of previous studies may be due to the insufficient statistical power, non-equivalent study designs, as well as different diagnostic classification methods.

The objective of our study is to investigate whether patients with RVO have a higher risk of developing AMI than controls using the “whole population” NHIRD in Taiwan, not “sample data” as reported in previous studies. Thus, we will have sufficient statistical power. Besides, during the study period, NHIRD adopted the International Classification of Diseases, Ninth Revision, Clinical Modification (ICD-9-CM) Codes, which are generally acknowledged worldwide. Therefore, the results of our study were compared with the results of previous studies. Furthermore, given the completeness of the NHIRD, we can have a long-term study period and can derived the risk ratio of developing AMI after controlling and adjusting for the impact of confounders.

## Materials and methods

### Study setting

Taiwan’s National Health Insurance (NHI) program, which was launched in 1995, currently covers the health care services of greater than 99% of Taiwan’s 23 million residents. [18] The National Health Insurance Research Database (NHIRD), which is maintained by the National Health Research Institutes of Taiwan, is a collection of all registration file data and claims data for all ambulatory and in-hospital patients in Taiwan. The identification of all patients in the database is encrypted before the data are released for research purpose. [19] Therefore, according to the rules of the Instituitional Review Board, writthen informed consent was waived. [19–20] Based on the healthcare claims of the entire population, we sought to compare the incidence rate of AMI in subjects with and without RVO during the 13-year period. This study was approved by the ethical committee of Yang-Ming University Hospital (2015A018).

### Inclusion and exclusion criteria

Using the Taiwan NHIRD from 1996 to 2013, we performed a retrospective cohort study. We first selected patients with RVO during January 1, 2001 to December 31, 2013. The diagnostic codes of RVO included central retinal vein occlusion (CRVO; ICD-9-CM codes 362.35) and branch retinal vein occlusion (BRVO; ICD-9-CM codes 362.36). Patients with RVO diagnoses from January 1, 1996 to December 31, 2000 were excluded to eliminate patients with previous, chronic RVO. The date of the first RVO claim was defined as the index date. We also randomly selected individuals who had never received a diagnosis of RVO as a comparison group. To eliminate the bias caused by the group differences, the RVO group and the comparison group were 1:3 matched using propensity score (PS) matching method, [21] in terms of age, gender, index year (the year of index date), use of antithrombotic drugs, obesity, stroke, hyperviscosity syndrome, and glaucoma. Both the RVO and comparison groups were tracked during the follow-up period to identify the occurrence of AMI (ICD-9-CM codes 410). Patients who received a diagnosis of AMI before the index date were excluded to ensure that patients with only newly diagnosed AMI were included.

### Statistical analysis

The demographic/clinical characteristics of the RVO and comparison groups were compared by chi-square test for categorical variables and two-sample *t*-test for continuous variables. A log-rank test was performed to describe and compare the cumulative hazard curves of AMI between the two groups. A multivariate Cox proportional hazard model was used to estimate the adjusted HRs for the occurrence of AMI. Covariates adjusted in the regression analysis were PS and relevant comorbidities, including diabetes, hypertension, hyperlipidemia, congestive heart failure, and chronic renal failure. [22] To eliminate the effect of the immortal time bias, these comorbidities were regarded as time-dependent variables. [23]

Furthermore, we divided the RVO group into BRVO and CRVO groups and compared the demographic/clinical characteristics between them. Finally we separately compared the HRs for AMI among the BRVO, CRVO and comparison groups. All statistical operations were performed using SAS statistical package, version 9.3 (SAS Institute, Cary, NC, USA).

## Results

### The study population

This study included 37921 patients with RVO and 113763 PS-matched comparisons. Among the patients with RVO, 11855 (31.3%) were diagnosed with CRVO, and 26066 (68.7%) were diagnosed with BRVO. Table 1 shows a summary of the characteristics of the RVO and the comparison groups. Because the two groups were well-matched on PS derived from age, gender, use of antithrombotic drugs, obesity, stroke, hyperviscosity syndrome, and glaucoma, no differences in theses variables and PS were found between the two groups. The mean age of the overall cohort was 62.4 years, with a standard deviation of 13.2 years. Greater than 60 percent of the subjects were over 60 years old. Males constituted a slightly higher proportion than females (51.1% vs. 48.9%). The mean follow-up period was 5.52 years in the RVO group and 5.55 years in the comparison group, without significant differences (*p* = 0.16). Comparing the prevalence of comorbidities in the RVO group with the comparison group, we identified significant differences in the prevalence of diabetes, hypertension, hyperlipidemia, congestive heart failure, and chronic renal failure (all *p* <0.0001). During the 13-year study period, AMI occurred in 1240 (3.22%) patients in the RVO group and in 2616 (2.3%) in the comparison group (*p*<0.0001).

**Table 1:** Characteristics of the study subjects.

Variable	RVO group n = 37921	Comparison group n = 113763	*p-*value
	n (%)	n (%)	
**Variables to generate PS**			
Age, year (mean±SD)	62.4±13.1	62.4±13.2	0.90
Age, categorical			>0.99
<50	6098 (16.1)	18302 (16.1)	
50–60	9038 (23.8)	27118 (23.8)	
60–70	10323 (27.2)	31003 (27.3)	
≥70	12462 (32.9)	37340 (32.8)	
Gender			>0.99
Male	19416 (51.2)	58249 (51.2)	
Female	18505 (48.8)	55514 (48.8)	
Antithrombotic drugs	9215 (24.3)	27752 (24.4)	0.71
Obesity	763 (2.0)	2396 (2.0)	0.78
Stroke	3992 (10.5)	11939 (10.5)	0.86
Hyperviscosity syndrome	429 (1.1)	1298 (1.1)	0.88
Glaucoma	3379 (8.9)	10077 (8.9)	0.75
**PS** (mean±SD)	0.124±0.102	0.124±0.102	>0.99
**Variables for adjustment**			
Diabetes	15020 (39.6)	35264 (31.0)	<0.0001
Hypertension	30194 (79.6)	69547 (61.1)	<0.0001
Hyperlipidemia	18852 (49.7)	45142 (39.7)	<0.0001
Congestive heart failure			<0.0001
Yes, with CHD	1499 (3.9)	3555 (3.1)	
Yes, without CHD	3743 (9.9)	9900 (8.7)	
No	32679 (86.2)	100308 (88.2)	
Chronic renal failure	4945 (13.0)	8208 (7.2)	<0.0001
**Follow-up period**, year (mean±SD)	5.52±3.56	5.55±3.66	0.16
**Incident AMI**	1240 (3.27)	2616 (2.30)	<0.0001

RVO: Retinal vein occlusion; PS: propensity score; CHD: coronary heart disease; AMI: acute myocardial infarction

### Cumulative hazard curves and log-rank test

**Fig 1:**
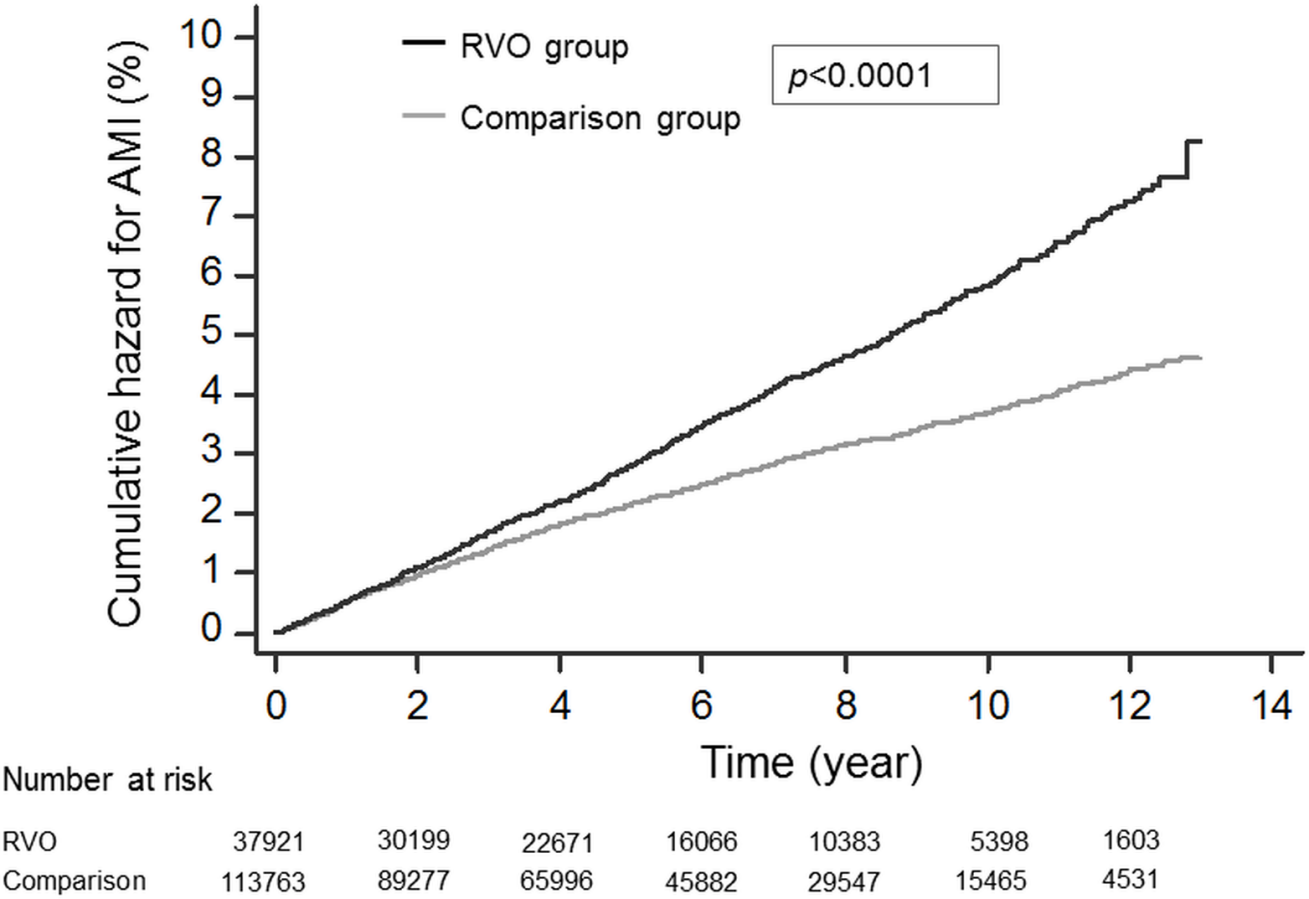
Cumulative hazard curves for AMI among the RVO and comparison groups. The black line represents the RVO group, and the gray line represents the comparison group. RVO: retinal vein occlusion.

Fig 1 illustrates the cumulative incidence curves of AMI in the RVO group and the comparison group. These two curves were moving apart from each other from the very beginning until the end of the study period. According to the log rank test, the RVO group had a significantly higher cumulative hazard of AMI than the comparison group (*p*-value<0.0001)

### HRs for AMI analyzed using Cox models

Table 2 displays the adjusted HRs for AMI during the 13-year study period calculated with multivariate Cox regression models. After adjusting for PS and confounders, the RVO group had a significantly higher hazard for AMI (adjusted HR = 1.21, 95% CI: 1.13 to 1.30). People with comorbidities, such as diabetes, hypertension, hyperlipidemia, congestive heart failure, and chronic renal failure, had a significantly increased risk of AMI (all *p*<0.0001). For those with both congestive heart failure and coronary heart disease (CHD, not AMI), the hazard of AMI was significantly increased 4.19-fold compared with those without congestive heart failure. For those with congestive heart failure but without CHD, the hazard for AMI was still statistically higher than those without congestive heart failure (adjusted HR = 1.96 with 95% CI: 1.80–2.14).

**Table 2:** Analyses of hazard ratios for AMI.

Predictive variables	Multivariate analysis	
	Adjusted HR (95% CI)	*p*-value
RVO (Yes vs. No)	1.21 (1.13–1.30)	<0.0001
Diabetes	1.31 (1.22–1.40)	<0.0001
Hypertension	1.74 (1.57–1.92)	<0.0001
Hyperlipidemia	1.37 (1.28–1.46)	<0.0001
Congestive heart failure		
Yes, with CHD	4.19 (3.82–4.59)	<0.0001
Yes, without CHD	1.96 (1.80–2.14)	<0.0001
No	Reference	
Chronic renal failure	1.23 (1.13–1.34)	<0.0001

AMI: acute myocardial infarction; HR: hazard ratio; CI: confidence interval; RVO: retinal vein occlusion; CHD: coronary heart disease

Propensity score and all the other variables in the table were included for adjustment in the Cox multivariate analysis

### Comparison of characteristics between the CRVO and BRVO groups

In Table 3, we divided the RVO group into the CRVO group (with 11855 patients) and BRVO group (with 26066 patients), and compared their demographic/clinical characteristics. The age distribution in the CRVO group was younger than that in the BRVO group. The CRVO group had a significantly higher proportions of hyperviscosity syndrome, glaucoma, and diabetes than the BRVO group. However, the two groups were similar in the proportions of obesity, stroke, and congestive heart failure. The two groups also had similar mean follow-up time (5.51 years in the CRVO group and 5.53 years in the BRVO group). The occurrence rate of AMI is 3.60% in the CRVO group, significantly higher than that of 3.12% in the BRVO group (*p*<0.0001).

**Table 3:** Characteristics of the CRVO and BRVO subjects.

Variable	CRVO group n = 11855	BRVO group n = 26066	*p-*value
	n (%)	n (%)	
**Variables to generate PS**			
Age, year (mean±SD)	61.3±14.5	62.8±12.4	<0.0001
Age, categorical			<0.0001
<50	2396 (20.2)	3702 (14.2)	
50–60	2656 (22.4)	6382 (24.5)	
60–70	2911 (24.6)	7412 (28.4)	
≥70	3892 (32.8)	8570 (32.9)	
Gender			<0.0001
Male	6367 (53.7)	13049 (50.1)	
Female	5488 (46.3)	13017 (49.9)	
Antithrombotic drugs	2782 (23.5)	6385 (24.5)	0.03
Obesity	218 (1.8)	443 (1.7)	0.35
Stroke	1223 (10.3)	2769 (10.6)	0.38
Hyperviscosity syndrome	176 (1.5)	253 (1.0)	<0.0001
Glaucoma	1600 (13.5)	1779 (6.8)	<0.0001
**PS** (mean±SD)	0.121±0.10	0.131±0.12	0.015
**Variables for adjustment**			
Diabetes	4964 (41.9)	10056 (38.6)	<0.0001
Hypertension	8934 (75.4)	21260 (81.6)	<0.0001
Hyperlipidemia	5713 (48.2)	13139 (50.4)	<0.0001
Congestive heart failure			0.67
Yes, with CHD	483 (4.1)	1016 (3.9)	
Yes, without CHD	1179 (10.0)	2564 (9.8)	
No	10193 (86.0)	22486 (86.3)	
Chronic renal failure	1854 (15.6)	3091 (11.9)	<0.0001
**Follow-up period**, year (mean±SD)	5.51±3.50	5.53±3.58	0.59
**Incident AMI**	427 (3.60)	813 (3.12)	<0.0001

CRVO: central retinal vein occlusion; BRVO: branch retinal vein occlusion; PS: propensity score; CHD: coronary heart disease; AMI: acute myocardial infarction

### Comparison of the risk of AMI between the CRVO and BRVO groups

**Fig 2:**
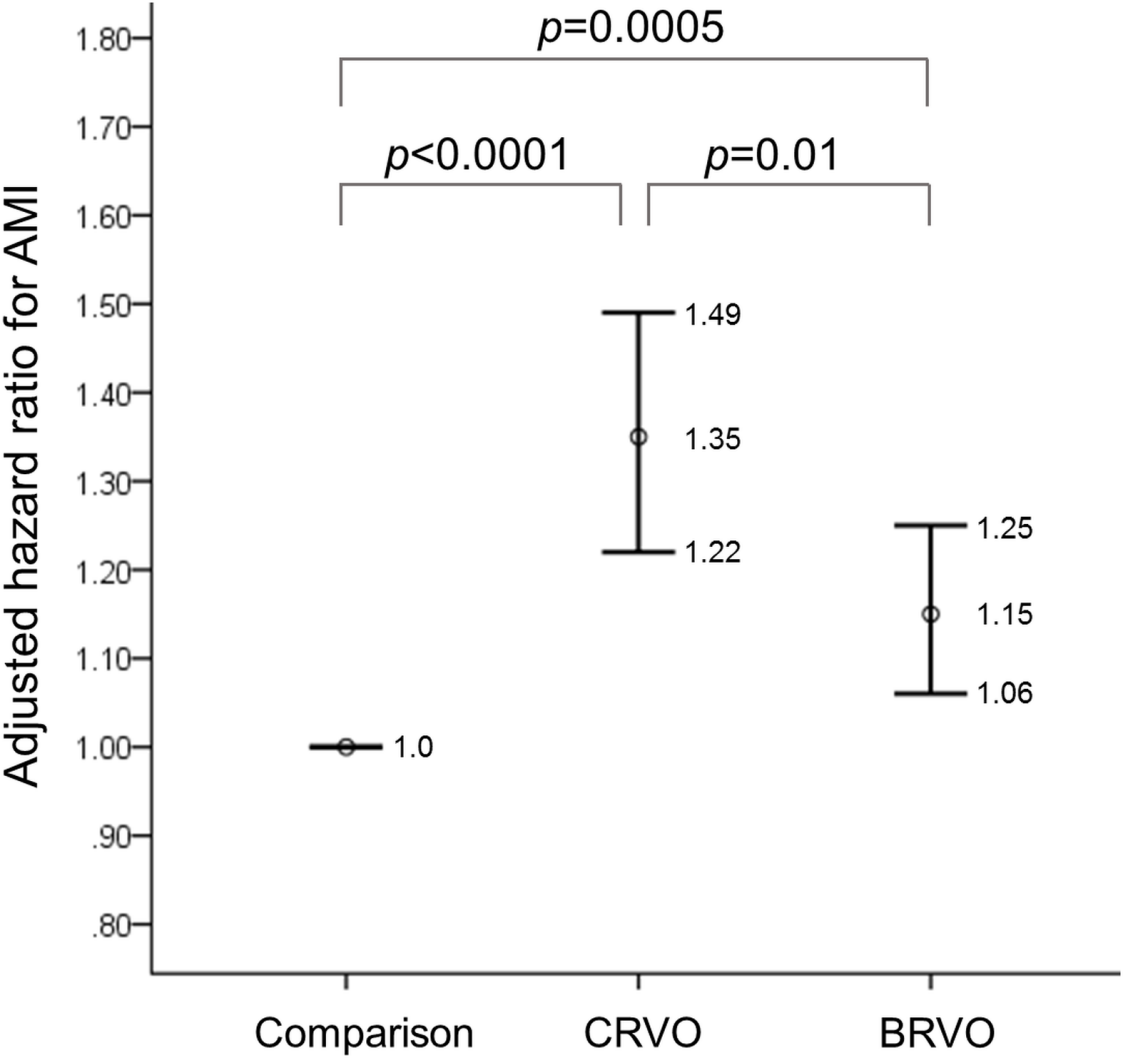
Hazard ratios for AMI in the BRVO and CRVO groups compared with the comparison group. AMI: acute myocardial infarction; BRVO: branch retinal vein occlusion; CRVO: central retinal vein occlusion.

Fig 2 compares the adjusted HR for AMI among the comparison, CRVO, and BRVO groups. Compared with the comparison group, the adjusted hazard ratio for AMI was 1.35 (95% CI: 1.22–1.49) in the CRVO group and 1.15 (95% CI: 1.06–1.25) in the BRVO group. When patients with BRVO and CRVO were compared, the CRVO group still had a significantly higher risk of developing AMI than the BRVO group (*p*-value = 0.01).

## Discussion

In this nationwide follow-up study using the complete population data from the Taiwan NHIRD, the risk of developing AMI were compared between 37921 RVO patients and 113763 PS-matched comparisons. After adjusting for PS and possible confounders, the RVO group had a significantly higher risk of developing AMI than the comparison group (adjusted HR = 1.21, 95% CI: 1.13–1.30). When RVO group was divided into CRVO and BRVO groups, we found both of them had significantly higher adjusted HR for AMI than the comparison group. And, the CRVO group had a significantly higher risk of developing AMI than the BRVO group (*p* = 0.01).

RVO, including CRVO and BRVO, is one of the most common causes of visual impairment, particularly in older people. [24] The pathogenesis of RVO has not been completely described, but occlusion resulting from thrombosis is a well-accepted theory. RVO mainly occurs at arteriovenous crossings where the retinal arteriole and venule share a common adventitia sheath. If the arterial wall is sclerotic, it may compress the underlying vein, and the turbulent blood flow in the vein will facilitate the formation of venous thrombosis and venous occlusion. [25] Similarly, the underlying mechanism of AMI involves a sclerotic artery (coronary artery), which causes thrombus formation and venous obstruction. [26] We postulate that RVO and AMI may be associated because the changes in retinal vessels may somehow imply that changes have also occurred in coronary vessels.

Another explanation for the association between RVO and AMI is that they have common risk factors. As noted in Table 1 in our study, the RVO group exhibits a greater proportion of systemic comorbidities, such as diabetes, hypertension, hyperlipidemia, congestive heart failure, and chronic renal failure, than the comparison group. The result is consistent with the results from previous studies. [9,10,12,16,25,27–31] Notably, these systemic comorbidities are also risk factors for AMI, thus potentially explaining why patients with RVO are predisposed to AMI.

As shown in Fig 1, the cumulative hazard of AMI in the RVO group was significantly higher than the comparison group. The result is compatible with the results of the population-based studies in the US and South Korea. [16,17] Hu’s study in Taiwan, with a follow-up time of 3 years, also revealed a higher cumulative incidence of AMI in the RVO group. [15] With a much larger sample size and a much longer follow-up period, our study further confirms the phenomenon.

Shih et al analyzed claims data from 10,081 patients with RVO aged 70 years in Taiwan and reported that patients with RVO had higher risks of subsequent myocardial infarction (HR = 1.29, 95% CI: 1.15–1.44). [9] Our study, analyzing data of the whole population, not only the elderly, also revealed a significantly higher adjusted HR for the patients with RVO than the individuals without RVO. Bertelsen et al reviewed the registries of 439 photographically verified patients with CRVO from four referral centers in Denmark and found that patients with RVO had a 1.9-fold increased risk of developing ischemic heart disease compared with the control cohort (HR = 1.9, 95% CI: 1.32–2.75). [14] However, in another study by Bertelsen et al utilizing the same data source, BRVO was not significantly associated with subsequent myocardial infarction (MI). [10] In our study, as shown in Fig 2, both the BRVO and CRVO groups have a significantly higher risk of developing AMI than the comparison group. In addition, the risk in patients with CRVO is significantly higher than the risk in BRVO patients. Thus, BRVO and CRVO may not have the same risk factors. [30] To the best of our knowledge, our study is the first to report a significantly higher risk of AMI in the CRVO group compared with the BRVO group.

One strength of our study is the use of the complete population database, precluding possible selection bias, and providing stronger statistical power. Another strength of our study is the accuracy of the diagnoses. In our healthcare system, patients must receive a standard protocol of examinations to confirm the diagnoses or the National Health Insurance Administration will not pay the fees for treatment. Previous studies also validated the accuracy of the diagnoses in the database. [32–34] Therefore, the diagnoses in our study, including RVO, AMI, and comorbidities, were thoroughly verified.

Another strength is the controlling and adjustment for possible confounders using PS matching method and PS analysis. In brief, PS is computed through mathematical algorithms from confounders that have impact on RVO, which means PS represents the overall impact of these confounders. Therefore, if the RVO and comparison groups are well-matched on PS (PS matching method), these confounders would be evenly distributed in the RVO and comparison groups, thus the PS matching method reduces the selection bias. PS analysis means that PS as well as other confounders are regarded as covariates in the regression adjustment, thus we can derive a more accurate relationship between RVO and AMI.

The limitation of our study is that some patients with asymptomatic RVO may not seek medication. Thus, subjects with undetected RVO were classified as the comparison group and not the RVO group. Such a misclassification bias would weaken the effect of RVO on the development of AMI. Even so, RVO patients in our study had a higher hazard of AMI. Therefore, our observation of an increased AMI risk in the RVO group is a real phenomenon.

It is a limitation that our database does not include tobacco use, which is a risk factor of RVO and AMI. However, we have included a wide range of variables to make our RVO and comparison groups balanced. Kolar P, [31] in a review article, has demonstrated that age, gender, obesity, stroke, hyperviscosity syndrome, and glaucoma are risk factors of RVO. In our study through PS matching method, these variables, as well as the use of antithrombotic drugs, have been well-matched and controlled between the RVO and comparison groups. Besides, risk factors that may confound the relationship between RVO and AMI, such as diabetes, hypertension, hyperlipidemia, congestive heart failure, and chronic renal failure, [31] have been adjusted under the multivariate regression adjustment.

Our study has clinical implications and public health implications. Clinically, our study reminds ophthalmologists to pay more attention to the risk of AMI when they are treating patients with RVO. In particular, patients with RVO who have risk factors for AMI, such as diabetes and hypertension, if are not under proper treatment, should be referred to physicians for early diagnosis and treatment to prevent the occurrence of AMI. From the public health perspective, policy makers are encouraged to enforce the surveillance for AMI risks in patients with RVO.
